# Monocular Visual-Inertial SLAM: Continuous Preintegration and Reliable Initialization

**DOI:** 10.3390/s17112613

**Published:** 2017-11-14

**Authors:** Yi Liu, Zhong Chen, Wenjuan Zheng, Hao Wang, Jianguo Liu

**Affiliations:** 1 National Key Laboratory of Science and Technology on Multi-Spectral Information Processing, School of Automation, Huazhong University of Science and Technology, Wuhan 430074, China; skyridermike@hust.edu.cn (Y.L.); jgliu@hust.edu.cn (J.L.); 2 Beijing Aerospace Automatic Control Institute, Beijing 100854, China; dingling721@126.com (W.Z.); whipraihust@163.com (H.W.)

**Keywords:** sensor fusion, SLAM, computer vision, inertial navigation, tightly coupled

## Abstract

In this paper, we propose a new visual-inertial Simultaneous Localization and Mapping (SLAM) algorithm. With the tightly coupled sensor fusion of a global shutter monocular camera and a low-cost Inertial Measurement Unit (IMU), this algorithm is able to achieve robust and real-time estimates of the sensor poses in unknown environment. To address the real-time visual-inertial fusion problem, we present a parallel framework with a novel IMU initialization method. Our algorithm also benefits from the novel IMU factor, the continuous preintegration method, the vision factor of directional error, the separability trick and the robust initialization criterion which can efficiently output reliable estimates in real-time on modern Central Processing Unit (CPU). Tremendous experiments also validate the proposed algorithm and prove it is comparable to the state-of-art method.

## 1. Introduction

Simultaneous Localization and Mapping (SLAM) has attracted a lot of attention from both robotic community and industrial community. Laser scanner was the primary sensor in earlier SLAM works (e.g., [1,2]). However, the size and weight of laser scanner significantly constrain the agility of the platform and thus the use of vision sensor gradually became a tendency [3,4,5,6,7,8]. The advantages of vision sensor include cheaper price, lighter weight and lower power consumption, which are essential to mobile platforms (e.g., Micro Aerial Vehicle). Furthermore, vision sensor has the capability for retrieving the environment’s appearance, color and texture such that it is possible to perform some high-level tasks such as scene recognition. While stereo camera [9,10,11], RGB-D camera [12,13] and omnidirectional camera sensors [14] have been proven suitable in some certain scenarios and applications, monocular SLAM provides a fundamental solution.

A typical SLAM system is composed of front-end and back-end. Front-end is in charge of performing data association for the back-end module. For visual SLAM, feature-based method and direct method are two main approaches in the front-end module. Direct method (e.g., [15,16,17]) directly uses the intensity values in the image to estimates the structure and motion, showing more robust than feature-based method in the texture-less scenarios. However, feature-based method is much less sensitive to exposure adjustment in video/image, and it may be a better choice for robust tracking under rich texture environment due to the invariance of descriptor. Back-end in SLAM is in charge of state inference after data association. From the viewpoint of the probabilistic framework, the purpose of back-end is to output the MAP (Maximum a posterior) estimates given the measurements from front-end. For this purpose, the back-end solutions have evolved from filter based approaches [3,18,19,20,21,22] to graph optimization methods [7,8,23]. The first real-time monocular SLAM system was presented by Davsion [3] with Extended Kalman Filter (EKF) framework, and Civera [18] improved its performance with the inverse depth feature parametrization. However, the maintaining of the dense covariance matrix in EKF is very expensive so that the size of features has to be very limited. Compared to filter-based methods, Graph optimization exploits the sparse structure and thus it enables fast computation by using sparse linear solvers. Current optimization solvers (e.g., g2o [24], Ceres [25], iSAM [26], GTSAM [27]) are able to solve a typical optimization problems with tens thousands of variables in few seconds. There also exists different strategies for combing the front-end and back-end. Klein [7] presented a novel parallel system. This real-time system consists of the tracking thread and the mapping thread. Motivated by the parallel design, Raul [23] presented an improved system with the concept of co-visibility graph for local mapping to efficiently keep the consistency for large scale environment. Forster [15] also utilized a parallel system by combining direct tracking for pose estimation and depth filter for feature estimation. Besides these methods, the sliding window strategy also shows good performance [11,28,29,30] and it keeps the computational time bounded by marginalizing out old states.

On the other hand, inertial measurement unit (IMU), as a complementary sensor to camera, is gradually used in the field of SLAM because it allows the recovery of the global roll, pitch and the undetermined scale in monocular SLAM. The early works of visual-inertial fusion were loosely coupled approaches [21,30,31,32] and then tightly-coupled approaches proved its superior performance that jointly optimize all state variables [11,33,34,35]. Among these tight fusion approaches mentioned above [11,20,29] are feature based approaches, which require the feature points that present a high degree of saliency. Mourikis [19] provided MSCKF algorithm and then consistency analysis of MSCKF was followed by [35,36], and [11,28,29] performed optimization framework by a sliding window to limit the computation. Forster [33] proposed the IMU preintegration on a manifold for sensor fusion and the iSAM back-end for incremental optimization. In contrast to these feature-based approaches, direct method fusion with inertial sensor provided by Alejo Concha [34] is the first work that combines the direct method with inertial fusion. Although direct methods are able to track features very efficiently, but they are more likely to fail due to exposure adjustment in vision camera sensor. From the viewpoint of computational complexity, the approaches based on sliding window like MSCKF can be thought as a constant-time solution for each visual frame, but they suffer from relatively larger drift since (a) the commonly used marginalization step usually leads to inconsistent estimates because the invariance is obeyed [35,37]; (b) the earlier observations are neglected. The work in [33] is done by an incremental optimization strategy (iSAM), but one of disadvantage is the unbounded complexity of memory, which can grow continuously over time.

**Figure 1:**
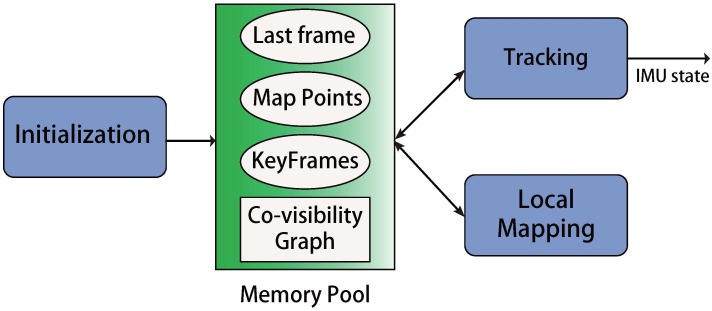
The global framework for our state estimation system. Note the Tracking and Local Mapping are two paralleled threads. A memory pool is utilized during the whole algorithm, which contains the states of map points, keyframes, and last frame. It also maintains the co-visibility graph for both the tracking and local mapping thread. Tracking thread uses the data from the memory pool to produce the state estimation in real-time. Meanwhile, the local mapping thread refines the data in the memory pool, which guarantees the estimation of tracking thread. Two threads perform different tasks and cooperate through the data in the memory pool. The memory pool will be initialized by the initialization process and will be described in Section 4. In the Section 3.1 and Section 3.2 we will discuss the tracking thread and the local mapping thread.

In this paper, we present a visual-inertial navigation system (VINS) that combines the visual SLAM approach and IMU preintegration technique [33,38] beyond the framework of ORB-SLAM [23] and PTAM [7]. Firstly, we derive a new IMU factor, motivated by the work in [35] with the corresponding preintegration method. The derivation is based on the continuous form which allows the use of high-order integration like Runge-Kutta. We stress that the derived IMU factor does not depend on the assumption that the IMU biases keep unchanged between two consequential keyframes such that our proposed IMU factor can better capture the correlation of state uncertainties. Thanks to the proposed IMU factor, given IMU poses (up to a scale) and the preintegrated measurements, we derive a linear least square formulation to initialize the system, which does not need to separately estimate the state variables. More important, since the proposed initialization method has considered the propagated uncertainty and the magnitude of the gravitational vector, we can have a robust mechanism to decide whether current information for initialization is enough or not, which is beyond the discuss in [38,39,40]. We then propose a well-designed parallel framework Figure 1 that runs a tracking thread and a local mapping thread at the same time. In the tracking thread, we only optimize the current IMU state with the IMU preintegration technique and the current vision factor for low computation cost. In the local mapping thread, we optimize all IMU states in the co-visibility graph G and all map points observed in the G together for a more consist map. For faster convergence, we employ the separability trick in the optimization that subtly uses the overlooked property–IMU velocity and biases are linear in the cost function of the proposed IMU factor.

The rest of the paper is organized as follows. Section 2 introduces the graph optimization used in estimation and the proposed IMU factor with the corresponding preintegration method. Section 3 presents our work for the tightly coupled approach for visual-inertial SLAM algorithm. Section 4 gives the principle of initialization for our monocular visual-inertial SLAM algorithm. Initialization scheme is by no means trivial for a monocular visual-inertial SLAM because initial feature depth and IMU biases can have significant effects on tightly-coupled SLAM system and the estimator usually suffers from the ill-conditioned cases (e.g., constant velocity). Notations: To simplify the presentation, the vector transpose operators are omitted for the case $A={\left[\begin{array}{c} a^{⊺},b^{⊺},\cdots ,c^{⊺} \end{array}\right]}^{⊺}$.

## 2. Graph Optimization

In this section, we adopt the formalism of factor graph [27] and derive a nonlinear least squares formulation to calculate the maximum a posterior (MAP) estimate of the visual-inertial state estimation problem.

### 2.1. IMU Factor with Preintegration

#### 2.1.1. IMU State and Motion Model

The IMU state to be estimated can be represented by a tuple, i.e.,

(1) $$\begin{array}{cc} X & =(R,p,v,b_{g},b_{a}) \end{array}$$

where $b:=(b_{g},b_{a})$, (R,p)∈SE(3) denotes the IMU pose in the global frame, $v:=\dot{p}\in R^{3}$ denotes the IMU velocity expressed in the global frame, $b_{g}\left(t\right)\in R^{3}$ denotes the gyroscope bias and $b_{a}\left(t\right)\in R^{3}$ denotes the accelerometer bias.

An IMU sensor consists of a 3-axis gyroscope and a 3-axis accelerometer. The gyroscope reading at the time *t* is corrupted by the bias and noise: $w\left(t\right)=\bar{w}\left(t\right)+b_{g}\left(t\right)+n_{g}$, where $\bar{w}\left(t\right)$ denotes the actual IMU angular velocity at the time *t*, $n_{g}$ is assumed to be a white Gaussian noise. Note that the effects form earth rotation is neglected. The accelerometer reading at the time *t* is also corrupted by the bias and noise: $a\left(t\right)=R^{⊺}\left(t\right)\left(\dot{v}-g\right)+b_{a}\left(t\right)+n_{a}$, where g is the gravity vector in the global frame and $n_{a}$ is also modeled as a white Gaussian noise.

Employing the IMU measurements model above and the random walk model for the time-varying IMU biases, we can easily conclude the IMU motion model in the following:

(2) $$\begin{array}{cc} \dot{X}= & f(X,u,n)\\ = & \left(RS\left(w-b_{g}-n_{g}\right),R\left(a-b_{a}-n_{a}\right)+g,v,n_{bg},n_{ba}\right) \end{array}$$

where the skew symmetric operator S(·) is given in Appendix, $u:=\left[\begin{array}{c} w,a \end{array}\right]$ are the measurements from IMU and $n:=\left[\begin{array}{c} n_{g},n_{a},n_{bg},n_{ba} \end{array}\right]$ are the white Gaussian noise with the known covariance $\Sigma \in R^{12\times 12}$.

#### 2.1.2. Mean Propagation

Ignoring the IMU noise n, we have a nominal IMU motion model:

(3) $$\dot{\hat{X}}=f\left(\hat{X},u,0\right)$$

where $\hat{X}$ denotes the nominal IMU state. Given the IMU state $X_{i}$ and the IMU measurements $u_{i:j}$ between the time step *i* and *j*, the predicted IMU state ${\hat{X}}_{j}$ at the time-step *j* can be recursively computed via the nominal motion model

(4) $$\begin{array}{cc} {\hat{X}}_{j} & =F(X_{i},u_{i:j}) \end{array}$$

Note that the transformation $F(\;\cdot \;,u_{i:j})$ above represents a series of integral operations and thus a naive implementation of computing $F(X,u_{i:j})$ is time-consuming and memory-occupied. Later we will provide a method to efficiently compute $F(X,u_{i:j})$ without need to re-integration.

#### 2.1.3. Error-State Motion Model

To concisely quantify the effects of IMU noise, we employ an error between the nominal IMU state $\hat{X}$ and the actual IMU state X motivated from [35]

(5) $$e:=\hat{X}⊖X:=\left[\begin{array}{c} \log(R^{⊺}\hat{R})\\ R^{⊺}\left(\hat{v}-v\right)\\ R^{⊺}\left(\hat{p}-p\right)\\ b_{g}-{\hat{b}}_{g}\\ b_{a}-{\hat{b}}_{a} \end{array}\right]\in R^{15}$$

Based on the nominal motion Equation (2) and the actual motion Equation (3) and the error Equation (5), we now can obtain the error-state propagation model:

(6) $$\dot{e}\approx Ae+Bn$$

where

(7) $$A=\left[\begin{array}{ccccc} -S(\hat{w}) & 0 & 0 & -I_{3} & 0\\ -S(\hat{a}) & -S(\hat{w}) & 0 & 0 & -I_{3}\\ 0 & I_{3} & -S(\hat{w}) & 0 & 0\\ 0 & 0 & 0 & 0 & 0\\ 0 & 0 & 0 & 0 & 0 \end{array}\right]$$

and

(8) $$\begin{array}{c} B=\left[\begin{array}{cccc} -I_{3} & 0 & 0 & 0\\ 0 & -I_{3} & 0 & 0\\ 0 & 0 & 0 & 0\\ 0 & 0 & I_{3} & 0\\ 0 & 0 & 0 & I_{3} \end{array}\right] \end{array}.$$

Note that the error-state propagation Equation (2) is almost an autonomous linear system, independent of state x. Therefore,

- The covariance P of e can be accurately computed by using the following differential equation:
  (9) $$\begin{array}{c} \dot{P}=AP+PA^{⊺}+B\Sigma B^{⊺} \end{array}$$
- The autonomous linear system can guarantee safe and reliable preintegration in the sense of the first-order approximation. Given $F(X_{i},u_{i:j})$, we can easily calculate $F(X,u_{i:j})$ based on the linear system theory for any X as the following:
  (10) $$F\left(X,u_{i:j}\right)=F\left(X_{i},u_{i:j}\right)⊕A\left(X⊖X_{i}\right)$$
  where ⊕ is the inverse of the operation ⊖ defined in (5):
  (11) $$X⊕e=(R\exp\left(e_{1}\right),v+Re_{2},p+Re_{3},b_{g}+e_{4},b_{a}+e_{5})$$
  The matrix $A\in R^{15\times 15}$ can be pre-integrated from the following differential equation
  (12) $$\dot{A}=AA$$
  with the initial state $A(t_{i})=I$. Here we stress that (10) makes hundreds of measurements $u_{i:j}$ unnecessary to be stored after preintegration.

The matrix A in (12) contains 225 elements. Fortunately, we can simplify the expression of A as the following:

(13) $$\begin{array}{c} A=\left[\begin{array}{ccccc} J_{1}^{⊺} & 0 & 0 & J_{4} & 0\\ -J_{1}^{⊺}S\left(J_{2}\right) & J_{1}^{⊺} & 0 & J_{5} & J_{4}\\ -J_{1}^{⊺}S\left(J_{3}\right) & -\Delta tJ_{1}^{⊺} & J_{1}^{⊺} & J_{6} & J_{7}\\ 0 & 0 & 0 & I_{3} & 0\\ 0 & 0 & 0 & 0 & I_{3} \end{array}\right] \end{array}$$

where $J_{1}\in \mathrm{SO}\left(3\right)$, $J_{2}\in R^{3}$, $J_{3}\in R^{3}$, $J_{i}\in R^{3\times 3}$ (i=4,5,6,7) can be preintegrated by the following differential equation:

(14) $$\begin{array}{cc} {\dot{J}}_{1} & =J_{1}S\left(\hat{w}\right),\;{\dot{J}}_{2}=J_{1}S\left(\hat{a}\right)\\ {\dot{J}}_{3} & =J_{2},\;{\dot{J}}_{4}=-S\left({\hat{w}}_{t}\right)J_{4}-I_{3}\\ {\dot{J}}_{5} & =-S\left(\hat{a}\right)J_{4}-S\left(\hat{w}\right)J_{5},\;{\dot{J}}_{6}=J_{5}-S\left(\hat{w}\right)J_{6}\\ {\dot{J}}_{7} & =J_{4}-S\left(\hat{w}\right)J_{7} \end{array}$$

with the initial guess $J_{1}\left(0\right)=I_{3}$ and $J_{i}\left(0\right)=0$ (i=2,⋯,7).

**Remark** **1.** Compared to the methods proposed in Forster [33] and HKST [30], our derivation is more straightforward and simple. Firstly, our proposed preintegration is born to be continuous. Secondly, unlike the preintegration of Forster and HKST, both of them fix the bias first when computing the 3 preintegration factors and simply use the first order Tyler expansion for the approximate Jacobian of IMU bias and IMU factor, our proposed IMU preintegration factor is based on the entire IMU state(including bias), use continuous differential equations which can better capture the correlations inside the IMU state. Thirdly, the defined error is invariant under the yaw angle transformation.

#### 2.1.4. IMU Factor

Given the IMU state $X_{i}$ and the IMU measurements $u_{i:j}$ between the time-step *i* and *j*, we have the predicted state ${\hat{X}}_{j}$ as presented in (4). According to the error definition (5) and the error-state motion model (6), we can get the uncertainty between the predicted state ${\hat{X}}_{j}$ and the actual state $X_{j}$

(15) $${\hat{X}}_{j}⊖X_{j}∼N\left(0,P_{ij}\right)$$

where the covariance matrix $P_{ij}$ is integrated from the differential equation (9) with the initial state P=0. In terms of factor graph, we have derived an IMU factor (i,j):

- **Connected Nodes:** the IMU state $X_{i}$ at time-step *i* and the IMU state the IMU state $X_{j}$ at time-step *j*.
- **Cost function:**
  (16) $$r\left(X_{i},X_{j}\right)=X_{j}⊖F\left(X_{i},u_{i:j}\right)\in R^{15}$$
- **Covariance matrix:** $P_{ij}$
- **Measurements:** the pre-integrated matrix A and the IMU biases $\left({\hat{b}}_{gi},{\hat{b}}_{ai}\right)$ used in the preintegration.

Then the proposed preintegration elements in (14) results in a closed-form solution of the predicted state $F(X_{i},u_{i:j})$ and therefore here we provide the closed form of the error function of the proposed IMU factor (16):

(17) $$\begin{array}{c} r\left(X_{i},X_{j}\right)=\left[\begin{array}{c} e_{r}+J_{r}^{-1}\left(e_{r}\right)J_{4}\left(b_{gi}-{\hat{b}}_{gi}\right)\\ R_{j}^{⊺}\left(v_{i}+g\Delta t_{ij}+R_{i}J_{2}-v_{j}\right)+J_{5}\left(b_{gi}-{\hat{b}}_{gi}\right)+J_{4}\left(b_{ai}-{\hat{b}}_{ai}\right)\\ R_{j}^{⊺}\left(p_{i}+v_{i}\Delta t_{ij}+\frac{1}{2}g\Delta t_{ij}^{2}+R_{i}J_{3}-p_{j}\right)+J_{6}\left(b_{gi}-{\hat{b}}_{gi}\right)+J_{7}\left(b_{ai}-{\hat{b}}_{ai}\right) \end{array}\right] \end{array}$$

where $e_{r}=\log\left(R_{j}^{⊺}R_{i}J_{1}\right)\in R^{3}$, $J_{r}\left(\;\cdot \;\right)$ and log(·) are given in Appendix. Note that the proposed error function is linear to all variables except $R_{i}$ and $R_{j}$. Later we will use this linear property to design the optimization algorithm.

### 2.2. Vision Factor

**Figure 2:**
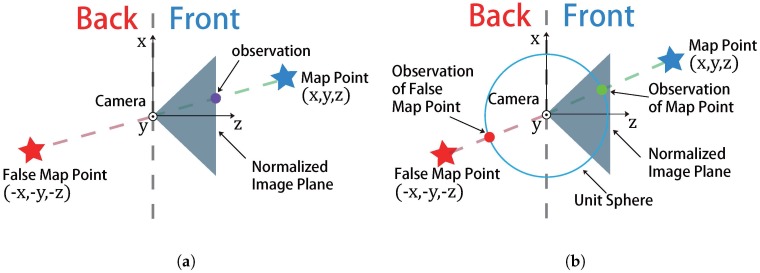
The difference between projection error and directional error. In projection error, since $\pi \left(x,y,z\right)={\left(x/z,y/z\right)}^{T}$ both the map point (in front of camera) and the false map point (in back of camera) would have the same observation, which can easily leads the algorithm falls into the local minimum. However the directional error employed by our algorithm, which normalized the direction vector between map point and camera center, can have different observations between the map points in the front and back, even their direction vectors is parallel with each other. (**a**) projection error employed by conventional vision factor; (**b**) directional error of our vision factor.

The conventional vision factor employs the re-projection error as the cost function, which is

(18) $$\pi (KR_{c}^{⊺}\left(f-p_{c}\right))-\mathrm{uv}$$

where $\pi \left(\;\cdot \;\right):R^{3}\to R^{2}$ is the projection function, $\left(R_{c},p_{c}\right)=\left(R,p\right)T_{IC}\in \mathrm{SE}\left(3\right)$ is the camera pose, K is the camera calibration matrix, $T_{IC}\in \mathrm{SE}\left(3\right)$ is the transformation from camera to IMU and $\mathrm{uv}\in R^{2}$ is the pixel observation for the map point $f\in R^{3}$. However, the zero re-projection error just implies that the predicted vector is parallel to the measured , which possibly results in a large number of local minimums. To alleviate this shortcoming, we employ the directional error as the cost function, resulting in a different vision factor. We present this improvement with more details in Figure 2.

- **Connected Nodes:** the IMU state $X_{i}$ at time-step *i* and the map point *f*
- **Cost function:**
  (19) $$g\left(X,f\right)=N\left(R_{c}^{⊺}\left(f-p_{c}\right)\right)-d\left(\mathrm{uv}\right).$$
  where $N\left(x\right)=\frac{x}{∥x∥}$ for $x\in R^{3}$ and $d\left(\mathrm{uv}\right)=N\left(K^{-1}\mathrm{UV}\right)$ ($\mathrm{UV}=\left[\begin{array}{c} \mathrm{uv},1 \end{array}\right]$). Note the directional error can be seen as the normalized vector between map point and camera center, which project the map point into a unit sphere. Thus, unlike the projection error in (18) which is an unbounded factor, the directional error is bounded into the range of [−2,2], which is friendly to the convergence of the algorithm.
- **Covariance matrix:** $\sigma I_{3}$

### 2.3. Nonlinear Least Squares Form

**Figure 3:**
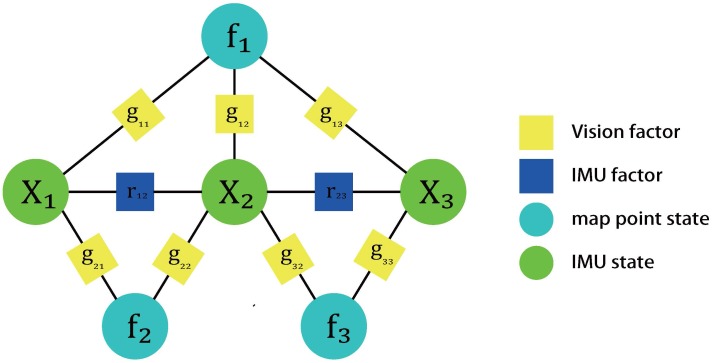
A VINS graph with 3 IMU states and 3 map points. The notation $X_{i}$ represents the IMU state at time-step *i*, $f_{i}$ represents the map point *i*. The notation $g_{ij}$ represents the vision factor and $r_{ij}$ stands for the IMU factor. Then the objective cost function for the VINS solution should be adding all factors ogether.

In our proposed system, optimization is used to correct the error due to sensor noise. Given all IMU measurements u and camera measurements z along the trajectory, the MAP estimate is

(20) $$\begin{array}{cc} X^{*} & =\arg\max_{X}p\left(X|z,u\right)\\ & =\arg\max_{X}\prod_{k}p\left(X_{k}|X_{k-1},u_{k-1:k}\right)\prod_{k,l}p\left(X_{k},f_{l}|z_{k,l}\right) \end{array}$$

where $X=\{X_{k},f_{l}\}$ includes the observed map points and the IMU states from time step 0 to *N*. Note that $p(X_{k}|X_{k-1},u_{k-1:k})$ and $p(X_{k},f_{l}|z_{k,l})$ correspond to the IMU factor and the vision factor, as discussed in Section 2.1 and Section 2.2. Based on the theory of factor graph, the optimization problem above can be abstracted into a graph (Figure 3) that consists of nodes ($X_{i}$ and $f_{l}$) and factors (r and g). The MAP estimate inference can be transformed into the following nonlinear least squares problem:

(21) $$\begin{array}{cc} X^{*}= & \arg\min_{X}\sum_{i}∥r\left(X_{i},X_{i+1}\right){∥}_{P_{i,i+1}^{-1}}^{2}+\sum_{i,l}{∥g\left(X_{i},f_{l}\right)∥}_{\sigma^{-1}I_{3}}^{2}\\ = & \arg\min_{X}{∥h\left(X\right)∥}^{2} \end{array}$$

Different from the standard least squares problem, the state space of the problem above is non-Euclidean space and thus we integrate the “lift-retraction” strategy into the conventional Gauss-Newton method for solving optimization, which has been summarized in Algorithm 1. Note that ⊞ in Algorithm 1 is *user-defined* and we choose

(22) $$\begin{array}{cc} X⊞\{\left(e_{i},e_{l}\right)\} & =\left\{\left(X_{i},f_{l}\right)\right\}⊞\left\{\left(e_{i},e_{l}\right)\right\}\\ & =\{\left(X_{i}⊕e_{i},f_{l}+e_{l}\right)\} \end{array}$$

where ⊕ is given in (11).
**Algorithm 1:** Solving Equation (21) by Using the Gauss-Newton Algorithm
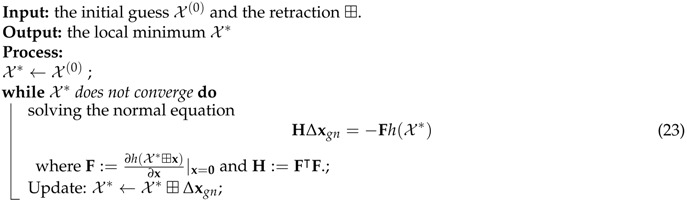


Optimization can provide a relatively accurate estimation for visual construction and the IMU state. However, the Cholesky decomposition used in solving the normal equation (23) suffers from the $O(\det{\left(\bar{H}\right)}^{3})$ complexity, where $\bar{H}$ has the same sparsity of H and only contains 1 and 0. To alleviate this, we present a novel way to solve (21), which employs the overlooked partial linear structure of (21) and the local observability of VINS. The related details will be given in Section 3.

## 3. Visual Inertial SLAM Algorithm

Our system is inspired by ORB-SLAM [23] which simultaneously runs the tracking thread and the local mapping thread in real-time.

### 3.1. Tracking

The tracking thread is in charge of estimating the latest IMU state, which involves twice optimization. In the first optimization, the initial value is given by the IMU preintegration. Then we search for the map points observation by 3D points’ projection. Finally, we perform the small-size optimization (24) which is solved by Algorithm 2. The optimization can be seen as an extension of the pose-only bundle adjustment in the ORB-SLAM [23]. Different with [23], the state variables brought by the IMU factor has been considered. We separate the state vector into two groups and optimized them separately. In the first step, we only update $R_{i}$ and $p_{i}$ thus we can avoid the large drift caused by the low-cost IMU sensor. Secondly, the optimization turns into a linear least squares problem w.r.t the $\left(v_{i},b_{gi},b_{ai}\right)$. We describe this solution with more mathematical detail in the Remark 2.

**Figure 4:**
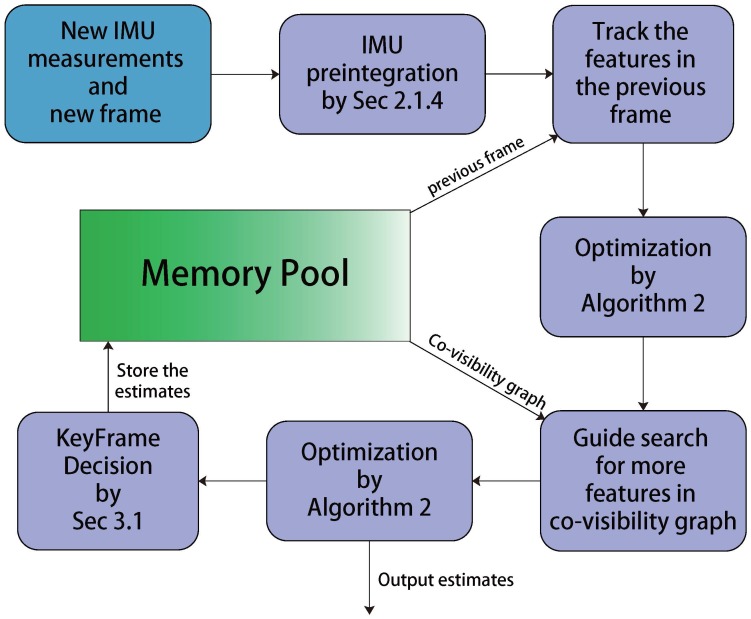
The framework of the Tracking thread.

After the first optimization, we perform a guided search of the map points from last frame. A wider search of the map points will be used if not enough matches are found. For efficient and robust data association, we use the projection method from the frames in the co-visibility graph to the current camera frame to perform feature correspondence. In order to keep the computational complexity bounded, we only deal with the keyframes in the local mapping thread. Therefore, there is a mechanism in the end of the threading thread that decides whether the current frame is a new keyframe. To insert a new keyframe, all the following conditions must be met: (1) More than 5 cm have been passed from the last keyframe; (2) More than 20 frames have been passed from last keyframe; (3) Current frame tracks at least 50 points and the number of common points between current frame and last keyframe should be less than 90% of last keyframe. The last condition ensures a visual change condition, and the 1st and 2nd condition will also reduce the number of unnecessary keyframes. We will also send a waiting signal to stop local mapping thread, so it can process the new keyframe as soon as possible. The framework of tracking is summarized in Figure 4.

**Remark** **2.** *To quickly output the estimate $x_{i}$, we employ a small-size optimization (24) instead of the full optimization:*

(24) $$\begin{array}{cc} x_{i}^{*}= & \arg\min_{x_{i}}{∥h\left(x_{i}\right)∥}^{2}\\ = & \arg\min_{x_{i}}∥r\left(X_{i-1},X_{i}\right){∥}_{P_{i-1,i}^{-1}}^{2}+\sum_{l}{∥g\left(X_{i},f_{l}\right)∥}_{\sigma^{-1}I_{3}}^{2} \end{array}$$

*where $x_{i-1}$ is the previous IMU state, $f_{l}$ denotes the map point observed in the current step. For efficient estimation, both $x_{i-1}$ and $f_{l}$ are fixed in (24). In addition, an ignorable property of (24) is that given the current pose $\left(R_{i},p_{i}\right)$, the optimization becomes linear least squares problem w.r.t. $\left(v_{i},b_{gi},b_{ai}\right)$. Therefore, we employ the* ***separability*** *trick for solving (24), which is summarized in Algorithm 2.*

**Algorithm 2:** Optimization (24) in Tracking
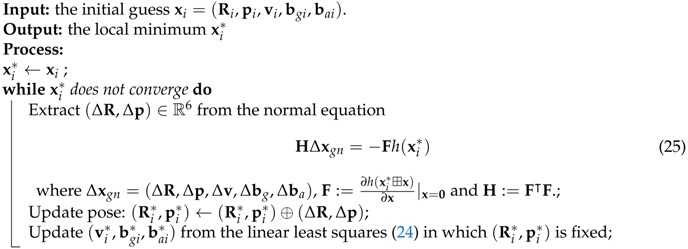


### 3.2. Local Mapping

**Figure 5:**
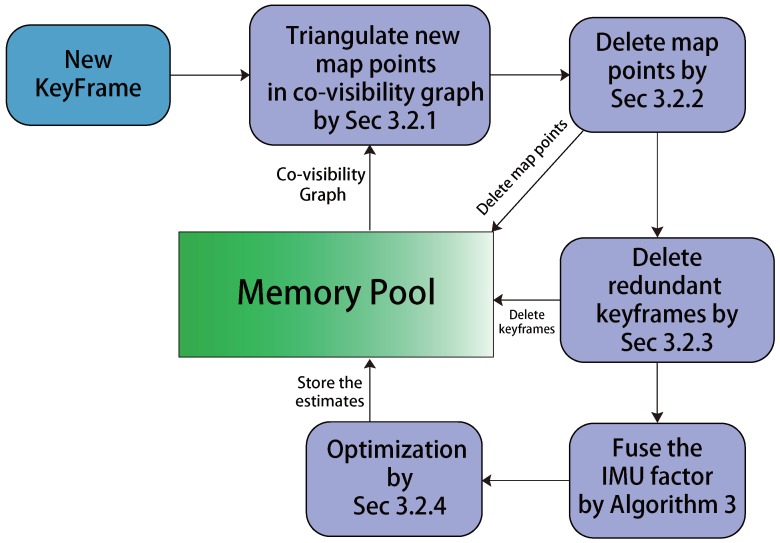
The framework of the Local Mapping thread.

**Figure 6:**
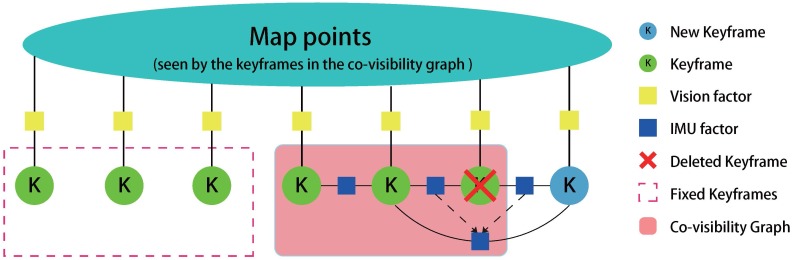
Graph illustration with 7 keyframes for the local mapping thread. Keyframes inside the co-visibility graph (red transparent area) will be connected by IMU factors, and their IMU state will be optimized by the local mapping thread. Other keyframes in the memory pool which also observe the map points will remain fixed (red dot rectangle). The graph also illustrates the IMU factor’s evolution when we delete a keyframe. Two IMU factors which are connected to the deleted keyframe will be fused into a new IMU factor by the Algorithm 3.

Once a keyframe is inserted from the tracking thread, the local mapping thread will begin its work that includes creating map points, deleting map points, deleting keyframes and performing optimization. The flowchart of the local mapping thread is presented in Figure 5, and we also add a graph to present the local mapping thread in Figure 6.

#### 3.2.1. Creat Map Points

When the local mapping thread gets a new keyframe, new map points observed in the new keyframe and the local keyframes will be created by triangulation. The following are the main steps. First, the projection method from the local keyframes is used to search the feature correspondences. The search is performed according to the time order, and it begins from last keyframe and stops once it fails to get a match. With the new feature correspondences from the search, we then calculate the coordinates of new map points by using the fast linear triangulation. In order to get rid of spurious data association, we only keep the new map points that are observed at least three times. Finally, the co-visibility graph will be updated by adding the undirected edges between the keyframes that share the same map points.

#### 3.2.2. Delete Map Points

Considering that outliers or incorrect feature correspondences will significantly affect the system performance, map points culling is needed before optimization. In the local mapping thread, we check the epipolar constraint and reprojection error of each map point for each keyframe which observes this point. In addition, we also check the parallax angle of each point. If the maximum parallax value is below a threshold, the map point will be removed. In this step, only the map points in the local map are processed.

#### 3.2.3. Delete KeyFrames

Deleting the redundant keyframes is beneficial for optimization, which saves the computational time. We discard keyframes whose 90% of map points have been seen in at least other three keyframes. When deleting a keyframe, we need to integrate two IMU factors (connected to this keyframe) into one IMU factor (connected to the last keyframe and the next keyframe). According to the theory of linear system, we derived an integration algorithm, summarized in Algorithm 3. Figure 6 shows the keyframe process in optimization graph, from which we can easily see the way of IMU factor fusion when delete a keyframe.
**Algorithm 3:** The Fusion of Two Consequential IMU Factors**Input**: two consequential IMU factors (i,j) and (j,k)
**Output**: IMU factor (i,k) **Process:** **Connected Nodes:** the IMU state $X_{i}$ and the IMU state the IMU state $X_{k}$. **Cost function:** (26)$$r\left(X_{i},X_{k}\right)=X_{k}⊖F\left(X_{i},u_{i:k}\right)\in R^{15}$$**Covariance matrix:**
$P_{ik}=A_{j,k}P_{ij}A_{j,k}^{⊺}+P_{jk}$. **Measurements:** the pre-integrated matrix $A_{ik}=A_{jk}A_{ij}$ and the IMU biases $\left({\hat{b}}_{gi},{\hat{b}}_{ai}\right)$. 


#### 3.2.4. Optimization

The last step of the local mapping thread is the optimization (21) with the nodes:

(1) the latest IMU state $x_{i}$ and all IMU states $x_{j}$ in the co-visibility graph (w.r.t. $x_{i}$);
(2) all map points $f_{l}$ observed by $x_{j}$ in the co-visibility graph (w.r.t. $x_{i}$);
(3) all IMU state $x_{k}$ that observes the map points in (b). Note that these variables are fixed in the optimization.

The involved factors are:

- The IMU factors that connects the consecutive IMU states in (a).
- The vision factors that connects the IMU states in (a) or (b) and the map points in (c).

To maintain a consistent estimate, we fix the IMU states in (b). Typically, the naive implementation of the optimization here suffers from the $O({\left(15n\right)}^{3})$ computational complexity in solving the *reduced* normal equation, where *n* is the number of IMU states in (a). Similar to the *separability* trick in Algorithm 2, we also use *separability* strategy on the optimization here so that the computational complexity can be reduced to $O({\left(6n\right)}^{3})$, which is given in the following Algorithm 4.

**Algorithm 4:** Optimization in Local Mapping
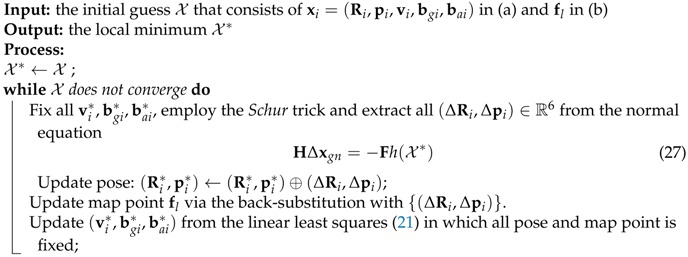


## 4. Initialization

**Figure 7:**
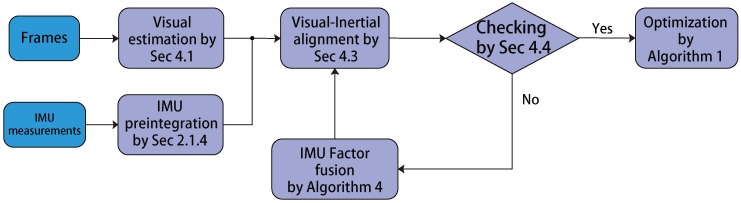
The framework of the initialization.

In this section, we propose a novel initialization method that provides a robust estimate at the beginning stage. The initialization is significant to the visual-inertial SLAM system due to the nonlinearity in optimization. Inspired by the linear property of the variables $\left(v_{i},b_{gi},b_{ai}\right)$ in the IMU factor, we propose a linear least square that can estimate the scale, the velocity, the IMU biases and their covariance matrix. To achieve the reliable estimates and handle the case of poor observability, the linear estimator will keep running until the uncertainty is lower than a threshold. The whole initialization scheme is presented by Figure 7.

### 4.1. Visual Estimation

At the first step, we employ the pure monocular ORB-SLAM [23] to produce the estimates of the frame and their IMU body poses. Note that the absolute scale *s* is unobservable in the pure visual odometry. The output $\left(R_{i},P_{i}\right)\in \mathrm{SE}\left(3\right)$ from the pure visual odometry is up to the scale *s*, i.e.,

(28) $$\left(R_{i},P_{i}\right)=\left(R_{i},\frac{p_{i}}{s}\right)$$

for i=0,1,⋯,N, where s∈R is the undetermined scale.

### 4.2. IMU Preintegration

Along with the visual estimation, we also perform the IMU preintegration as shown in Section 2.1. This step will output *N* IMU factors: the IMU factors (0,1), (1,2), ⋯, (N−1,N). Note that here the nominal IMU biases used for the preintegration of (12) and (9) are zeros.

### 4.3. Visual-Inertial Alignment

After visual estimation and IMU preintegration, we perform the visual-inertial alignment to roughly estimate the scale, gravity, velocity, IMU biases. First of all, we substitute (28) into the IMU cost function $r(X_{i-1},X_{i})$ and then we can see that the variables *s*, g, $\left(v_{i},b_{gi},b_{ai}\right)$ and $\left(v_{i-1},b_{g,i-1},b_{a,i-1}\right)$ are linear in this the term $r(X_{i-1},X_{i})$. Fixing the variables $\left(R_{i},P_{i}\right)$ for i=0,1,⋯,N, the MAP problem from (21) becomes

(29) $$\begin{array}{cc} X^{*}= & \arg\min_{X}\sum_{i}{∥r\left(X_{i},X_{i+1}\right)∥}_{P_{i,i+1}^{-1}}^{2}\\ = & \arg\min_{X}{∥\bar{h}\left(X\right)∥}^{2} \end{array}$$

where $X=(s,g,v_{0},b_{g0},b_{a0},\cdots ,v_{N},b_{gN},b_{aN})$. Note that here $\bar{h}\left(X\right)$ is almost linear to the variable block *X*. Thus we can straightforwardly obtain the solution $X^{*}$ of (29) using the linear least square. However, the linear solution for (29) does not consider the magnitude ∥g∥=9.8 and thus it easily gets ill-conditioned. If we consider the magnitude constraint of g, the linear optimization (29) becomes

(30) $$\begin{array}{cc} & \min_{X}{∥\bar{h}\left(X\right)∥}^{2}\\ \mathrm{st}: & g^{⊺}g=9.8^{2} \end{array}$$

The new optimization problem (30) is quadratically constrained quadratic program (QCQP) problem, which is a convex problem. It is well known that the local minimum in convex optimization is always a global minimum. Thus we convert (30) to a equivalent unconstrained form formulated in factor graph

(31) $$\begin{array}{c} \min_{X}{∥\bar{h}\left(X\right)∥}^{2} \end{array}$$

with a corresponding retraction

(32) $$X⊕e=(s+e_{s},\exp\left(Ce_{g}\right)g,v_{0}+e_{v0},b_{g0}+e_{bg0},b_{ba0}+e_{ba0},\cdots ,b_{aN}+e_{baN})$$

where $e=\left[\begin{array}{c} e_{s},e_{g},e_{v0},e_{bg0},e_{ba0},\cdots ,e_{vN},e_{bgN},e_{baN} \end{array}\right]\in R^{9N+12}$, $e_{g}\in R^{2}$ and $C\in R^{3\times 2}$ can be regarded as the null space of g. The use of (32) can grantee that the magnitude of g keeps unchanged after optimization.

### 4.4. Checking

It is well-known that a good visual-inertial alignment requires sufficiently motion. For robust estimates, we expect a smart checking step that is in charge of deciding if the estimate $X^{*}$ from last step (Section 4.3) is safe or not. Here we adopt a value to quantify the accuracy/uncertainty of the estimate $X^{*}$, which is the worst-case estimation error [41,42]

(33) $$\lambda_{max}\left({\bar{H}}^{-1}\right)$$

where $\bar{H}$ is the information matrix of scale and gravity, extracted from the Hessian matrix for the optimization problem (31), evaluated at the point $X^{*}$. Note that the larger value of $\lambda_{max}\left({\bar{H}}^{-1}\right)$, larger uncertainty of gravity and scale.

### 4.5. Optimization

If $\lambda_{max}\left({\bar{H}}^{-1}\right)$ is more than a threshold $\sigma_{int}$, the system will accept the estimate $X^{*}$. To refine the estimate $X^{*}$, we perform the optimization process (21) with all IMU preintegration and visual measurements. After this step, we have finished the whole initialization.

### 4.6. IMU Factor Fusion

If $\lambda_{max}\left({\bar{H}}^{-1}\right)$ is less than the threshold $\sigma_{int}$, the system will reject the estimate $X^{*}$ and wait a time-step for reinitialization. The reinitialization will be boosted with all measurements from time-step 0 to time-step N+1. Before the reinitialization, we perform IMU fusion of the IMU factors (N−2,N−1) and (N−1,N) to bound the size of the IMU factors. Note that the fusion algorithm is given in Algorithm 3.

## 5. Implementation Details and Results

**Figure 8:**
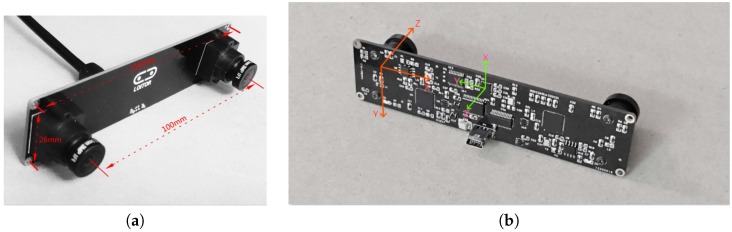
Loitor Sensor and coordinate system of IMU and left camera. Note we only use the left camera and the IMU sensor for testing our monocular visual inertial SLAM. For more details about the Loitor Sensor, see Loitor’s SDK page: https://github.com/loitor-vis. (**a**) Loitor Sensor; (**b**) The coordinate systems of IMU sensor and left eye camera.

The algorithm is implemented via C++11 code with ceres-solver [25] for nonlinear optimization framework. The proposed method runs in real-time (20 Hz) for all experiments on a standard computer (Intel Pentium CPU G840, 2.8 GHz, Dual-Core, 8 GB RAM). We test and evaluate our monocular visual-inertial SLAM system in both the low-cost, off-the-shelf visual-inertial sensor (Figure 8) and the EuRoC dataset [43].

At the beginning of the tracking thread (in Section 3.1), we select keypoints that are well-distributed in the current image. First we detect FAST corners in the 4 pyramid levels of the image. We then split the image into the 32×32 blocks. For each block, we calculate the average Shi-Tomasi score [44] for the FAST corners inside this block. Then we filter out those FAST corners below a specific threshold and calculate the number of the rest FAST corners in each block. If there is a block in which the number of FAST corners is very small (below the 20 percent of the median value of all blocks), we set the threshold to be half of the original one to get more FAST corners. If there is a block that does not contain any FAST corner, we split the image into the 16×16 blocks and repeat the selection steps above. After extracting the FAST corners, we then calculate the orientation and ORB descriptor for each retained corner. This stage takes about 17 ms on our computer.

After obtaining these keypoints with descriptors, we use the preintegrated IMU measurements (Section 2.1) to get the initial guess of the IMU state (1). In order to deal with the extreme case for the low-cost accelerometer, we filter out those accelerometer readings that are more than 50 times of the last reading. Then we start to perform the guided search of map points in the tracking thread (Section 3.1). Note that the feature correspondences in this step is coupled with the invariance property of the ORB descriptor such that the keypoints in the current frame can be matched with some earlier observations. In addition, we use the efficient subspace dog-leg algorithm in ceres-solver [25] to implement the nonlinear optimization (24). Multi-threads to compute the cost functions and jacobians are used to speed up the system.

We pay more attention about the outliers in the local mapping thread (Section 3.2). In order to gain robust performance, huber loss function with the scale value of 0.2 is used in the vision factors. To get rid of the effects caused by outliers, we first optimize with the huber loss function and then delete the vision factors whose cost function value is more than 0.2. We also check the estimated depth between each map points and keyframes. Map points with negative depth value will be seen as outliers and deleted. After deleting those map points that are outliers, we perform the nonlinear optimization without huber loss function.

### 5.1. Initilization Implementation

**Figure 9:**
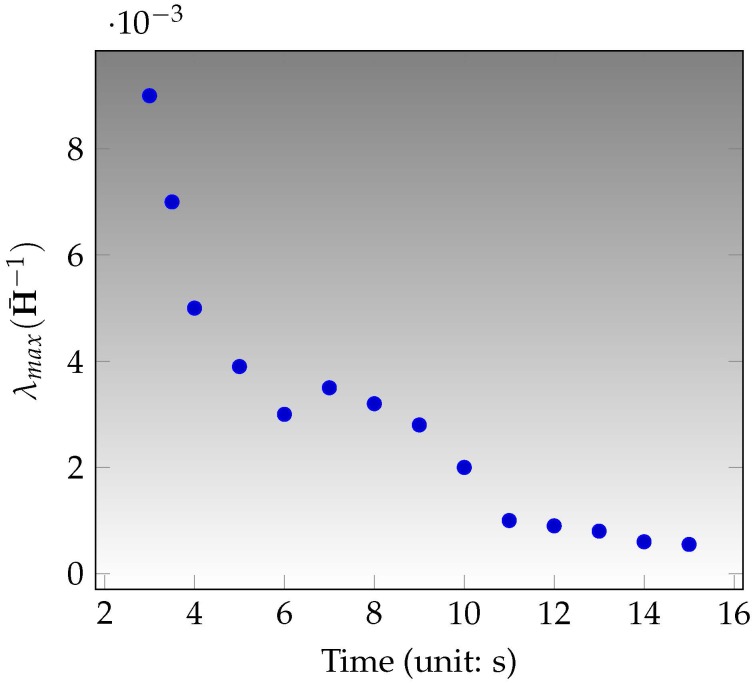
IMU initialization in V1_01_easy: the uncertainty of gravity and scale.

The proposed VINS initialization is evaluated in the in the sequence V1_01_easy. Because the robust estimates from visual odometry also need enough information, we perform the SE(3) estimates of the IMU poses at the first 3 s with the visual initialization from ORB-SLAM [23] and then implement the initialization method presented in last section. Figure 9 shows the uncertainties of gravity and scale, which converges after 11 s. The convergence means that the information is enough and then the system can work with a reliable initial estimate. The novelty of our method is

- Our method jointly optimizes the scale, gravitational vector, IMU biases, IMU velocity with proper covariance matrix from preintegration.
- Our method subtly uses the knowledge of the magnitude of the gravitational vector such that the ambiguity between the gravitational vector and the accelerometer bias can be avoided.
- We have a criterion to check whether the estimates for initialization is robust or not.

Since the proposed initialization method is convex which means a unique minimum solution, we optimize the intialization with Gauss Newton method for faster convergence. The Gauss Newton method is implemented by our own source code with Eigen C++ library [45]. The time cost for this optimization in initialization method is 23 ms on average. Note here we do not use huber loss function cause there is no outlier in IMU measurements. Neither ransc nor multi-thread implementation is needed. After this initialization module, we scale the poses of cameras and the positions of the map points.

### 5.2. Preliminary Test on Low-Cost Hardware

In this subsection, the adopted visual-inertial sensor is the Loitor inertial Stereo camera which is a low-cost device. The Loitor device contains a synchronized global shutter stereo camera which is able to output the 640×480 images at the frequency 30 Hz. The device also includes a MPU-6050 IMU with the frequency 200 Hz. The stereo camera and the IMU sensor have been synchronized. This sensor is calibrated by the calibration toolbox Kalibr [46]. Note here although the device contains a stereo camera, we just use the output of the left camera for testing our system. The entire algorithm is implemented in C++ using ROS for acquiring device data.

**Figure 10:**
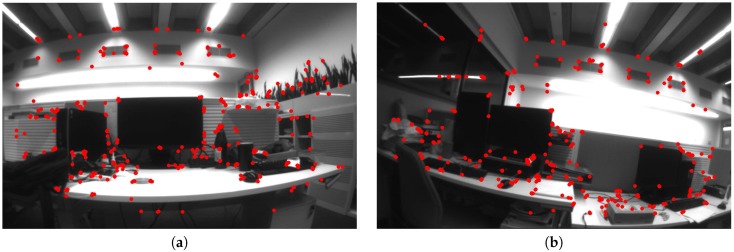

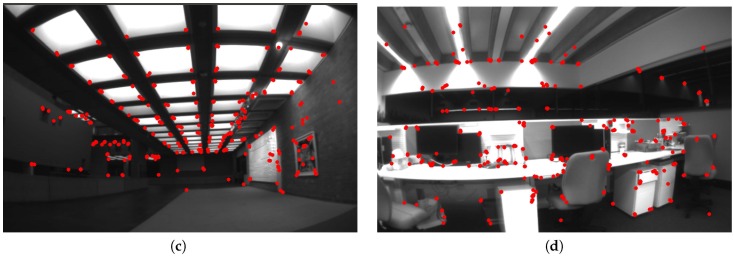
The distribution of keypoints in the images. (**a**,**b**,**d**) are taken in computer rooms and (**c**) is taken in the meeting hall.

**Figure 11:**
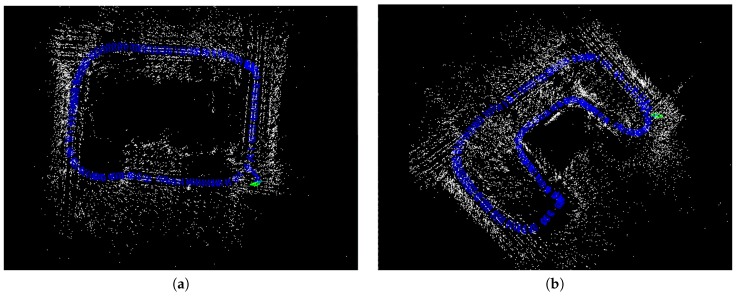
The top view of two estimated trajectories (in blue triangle) and map points (in white points) of our system. We can see the trajectory in (**a**) has some drift at the green point. Drift also exists in the image (**b**), but is not obvious. Both the experiments are implemented under an indoor environment with the size of 60 m × 60 m.

The algorithm is tested under an indoor scene with random texture. Chess board or any special visual tag is unavailable. The rate of the algorithm is 20 Hz on our computer, with a hand-held Loitor device. Figure 10 shows the well-distributed keypoints in the images. The top view of the estimated trajectory from the proposed system is plotted in Figure 11.

### 5.3. Evaluation on EuRoC

**Figure 12:**
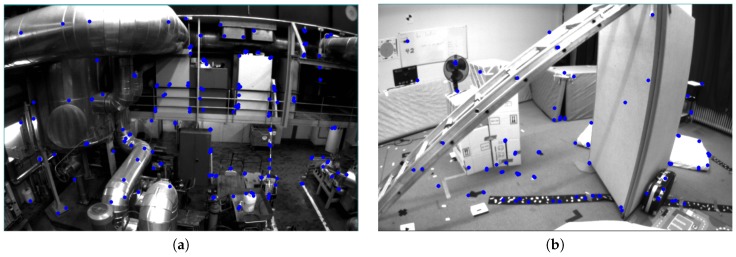
The different rooms in EuRoC data sets. Data sequences of Machine Hall in (**a**) have rich texture while Vicon Room of (**b**) have a lot of white walls which make them difficult for feature tracking.

The accuracy of our Visual-Inertial SLAM is evaluated in the 11 sequences of the EuRoC dataset. The EuRoc dataset provides synchronized global shutter WVGA stereo images at 20 Hz with MEMS IMU measurements at 200 Hz and trajectory ground-truth under different rooms in Figure 12. The dataset was collected by a MAV and ground truth is gained by a Vicon motion capture system which provided 6 DOF(degree of freedom) pose measurements at 100 Hz of a coordinate frame. For more detail we refer to paper [43].

**Figure 13:**
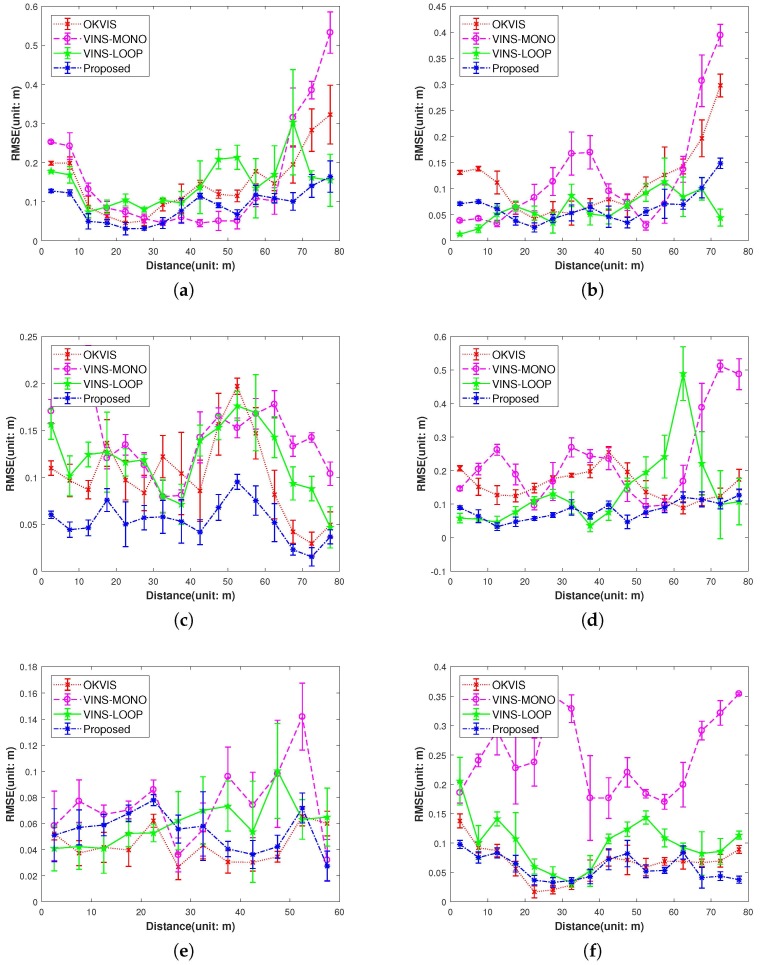

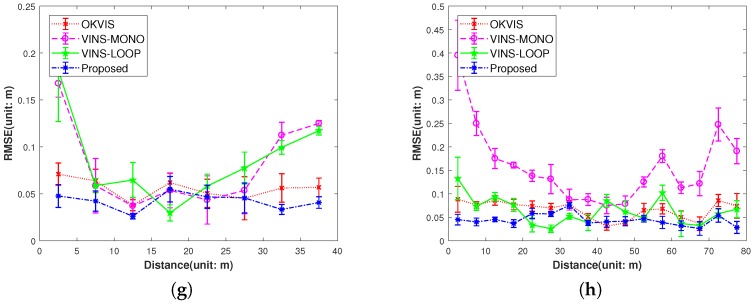
Comparison of the proposed method versus the OKVIS, VINS-MONO and VINS with loop closure (VINS-LOOP). The OKVIS uses a stereo visual-inertial sensor and the VINS-MONO (VINS-LOOP) uses a monocular visual-inertial sensor. Our algorithm has substantial improvement over other two methods in the Machine Hall (MH01-MH04) data sequences, also has comparable result with OKVIS in the rest of data sequences. Note we haven’t show the results of V103 and V203, since our algorithm fails to run the V103 and OKVIS fails to run the V203 data sequence. (**a**) MH_01_easy; (**b**) MH_02_medium; (**c**) MH_03_medium; (**d**) MH_04_difficult; (**e**) V1_01_easy; (**f**) V1_02_medium; (**g**) V2_01_easy; (**h**) V2_02_medium.

**Figure 14:**
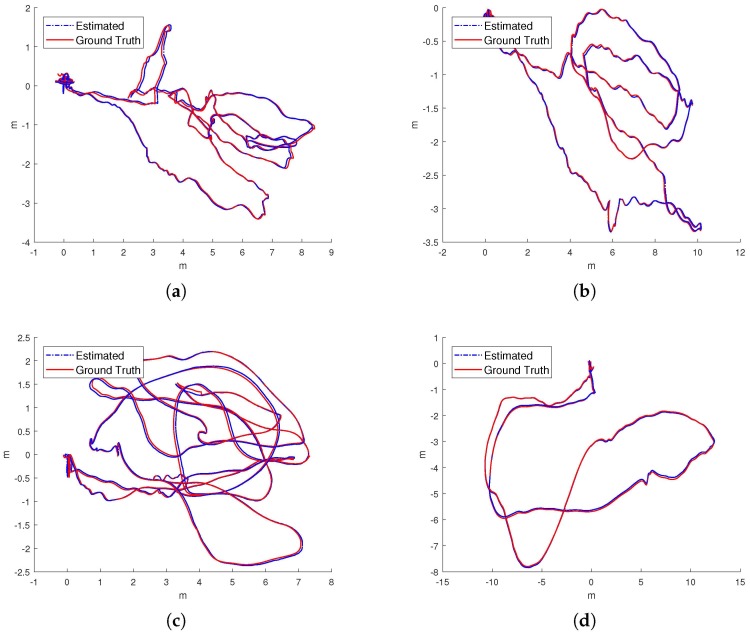

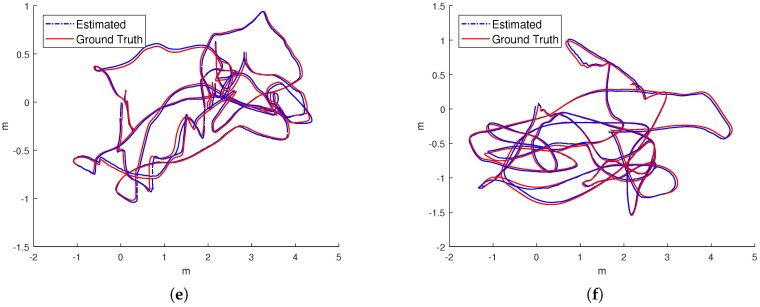
The top-views of estimated trajectory from our proposed approach (blue line) and ground truth (red line) of the dataset. (**a**) MH_01_easy; (**b**) MH_02_medium; (**c**) MH_03_medium; (**d**) MH_04_difficult; (**e**) V1_01_easy; (**f**) V1_02_medium.

IMU initialization is performed inside the SLAM system. Our system fails to run the sequence V1_03_difficult since the visual only SLAM failed to initialize under the extreme movement. For other data sequence, our SLAM algorithm can run in real-time without tracking lost. Table 1 presents the results of RMSE and standard deviation (in terms of translation) for different data sequences in EuRoC dataset. We evaluate the trajectories through the ATE method [47] which align the trajectories first, and then group them by the distance, finally compute the RMSE for each group. We present more details for the comparisons in Figure 13. In Figure 14 we plots some trajectories for our SLAM estimations and the ground truth (in top view).

We compare our proposed system with two state-of-art reasearch: stereo-inertial odometry OKVIS [11] and the VINS-MONO [30] without loop-closure and VINS-MONO with loop closure for completeness. Figure 13 shows the results of three system in terms of RMSE. From the error bar results in (b), (d), (e) and (f) in Figure 13, we can see our algorithm significantly outperforms the state-of-art algorithm VINS-MONO [30] and OKVIS [11] with stereo camera, which can be explained by the following:

- Our proposed IMU factor is more linear and it does not need reintegration when optimization. The cost function of our proposed IMU factor is more linear in terms of the defined retraction ⊕. The propagated covariance can better reflect the uncertainty of the physical system.
- The use of the separability trick and the novel vision factor makes convergence faster than the conventional method such that local or global minimum can be reached after few iterations in optimization.
- The use of co-visibility graph in our system can provide edges from current IMU state to the map points observed by the earlier IMU states, Since the data sequences in EuRoC is taken in a single small room, the drone can get the earlier observations easily by turning around, which makes the algorithm with co-visibility graph performs with much better precision.
- The fusion of IMU factors also provides the constraints between two consequential IMU states.

We would let readers know that although our algorithm performs with high precision, it fails to run the V103 data sequence. This data sequence has extreme movement at the beginning which is the main reason for the initialization failure in our system. In comparison, OKVIS [11] with a stereo camera can run without performing initialization which make the algorithm handling this data sequence easily. However, OKVIS fails to process the V203 data sequences. On the other hand, VINS-MONO [30], use sparse optical flow tracking as an independent front-end module to retrieve data association. Optical flow is a robust way for tracking features in video, which makes the initialization successfully and let the algorithm can process all the data sequences in EuRoC dataset. However, the algorithm suffers from low precision for ignoring the early observations. Besides, optical flow is not accurate for feature tracking in sub-pixel accuracy.

## 6. Discussion and Future Work

In this paper, based on the pure monocular vision ORB-SLAM [23], we present a monocular visual-inertial SLAM system with our new IMU factor, vision factor, initialization. The proposed visual-inertial slam has high precision over the EuRoC dataset. One of the main reason behind this, is the co-visibility graph we employed from the ORB-SLAM [23], since even we found even the stereo ORB-SLAM [23] without IMU can have higher precision than OKVIS [11] with stereo-inertial sensor. The co-visibility graph makes it possible to utilize the early observations while the sliding window based methods ignore them. Our algorithm has substantial improvement on Machine Hall data sequences in EuRoC, since the visual texture is very friendly for ORB feature tracking and co-visibility graph construction. Meanwhile, our algorithm achieves the comparable performance with the state-of-art method OKVIS which use a stereo camera and IMU sensor on the Vicon Room data sequences. In these data sequences we found our algorithm happened to track lost for a few times since they contain a lot of white walls and gray boards which is hard to detect any features on them. We can easily see this effect in Figure 12.

Since the algorithm still use the same front-end module, same keyframe decision with visual ORB-SLAM [23], we still think there is lots of things to do for further improvement. (1) For the front-end, we think direct method, which directly use the gray scale value into optimization, is a promising way since it can use weak feature that just have gradient value. Like in the DSO algorithm [17], by understanding more exposure adjustment in optical camera, the algorithm have surprising precision with impressive robustness. (2) For the vision factor in back-end, we found that our implementation of directional error have higher precision, but it is a bit slower than original bundle adjustment. There will be more comparison with more details between direction and projection vision factor in our future research. Also, we think getting rid of the map points that their parallax are below certain threshold is not reasonable for it lost the rotation information given by those map points. However, map points with low parallax will turns the system into ill-posed since their location is not observable. Therefore, developing a vision factor that can make use of low parallax is essential. (3) Some basic technique like on-line calibration for the sensor, loop closure can also be added into system. Machine learning methods that detect movable objects in visual observation can also be tried.

## 7. Conclusions

This paper demonstrates a new method for the monocular vision and inertial state estimation algorithm with a real-time implementation. The proposed IMU preintegration not only reaches the state of art efficiency, but also have better linear form which can better capture the correlation of state uncertanties. To increase the speed of the algorithm, the separability trick and the novel vision factor for fast computation was used in both the tracking thread and the local-mapping thread. Thanks to the proposed IMU preintegration with better linearity, the proper weight and the reasonable criterion to check the reliability of the estimates, our initialization method is fast and reliable, which solves a convex optimization with less uncertainty. So far we have build a tightly coupled visual-inertial SLAM system that can run with real-time performance in unknown environment. The future work will be mainly on providing more data to give more insights about the performance of our initialization and seek a better model for the tightly-coupled visual-inertial SLAM’s back-end. 

## Figures and Tables

**Table 1:** RMSE and Std deviation results for data sequence.

Sequence	RMSE (Unit: m)	Std (Unit: m)
V1_01_easy	0.0542	0.0194
V1_02_medium	0.0607	0.0246
V1_03_difficult	X	X
V2_01_easy	0.0424	0.0145
V2_02_medium	0.0430	0.0150
MH_01_easy	0.1010	0.0459
MH_02_medium	0.0643	0.0294
MH_03_medium	0.0632	0.0257
MH_04_difficult	0.0921	0.0384
MH_05_difficult	0.1378	0.0348

## Appendix A. Math

Here we provide the mathematical functions used in this paper. For more details, see [48]. The operator S(·) transforms a 3-dimensional vector to a 3×3 matrix:

(A1) $$S\left(x\right)=\left[\begin{array}{ccc} 0 & -x_{2} & x_{3}\\ x_{2} & 0 & -x_{1}\\ -x_{3} & x_{1} & 0 \end{array}\right]$$

for $x={\left[\begin{array}{c} x_{1},x_{2},x_{3} \end{array}\right]}^{⊺}$. The exponential mapping exp(·) transforms a 3-dimensional vector to a 3×3 rotation matrix:

(A2) $$\begin{array}{c} \exp\left(x\right)=I_{3}+\frac{\sin(∥x∥)}{∥x∥}S\left(x\right)+\frac{1-\cos(∥x∥)}{{∥x∥}^{2}}S^{2}\left(x\right) \end{array}$$

for $x\in R^{3}$. The logarithm mapping: for R∈SO(3)

(A3) $$\begin{array}{c} \log\left(R\right)=S^{-1}\left(\frac{\theta (R-R^{⊺})}{2\sin\theta }\right) \end{array}$$

where $\theta =\arccos(\frac{\mathrm{tr}(R)-1}{2})$. The right Jaocbian: for $x\left(\neq 0\right)\in R^{3}$

(A4) $$\begin{array}{cc} J_{r}\left(x\right) & =I_{3}-\frac{1-\cos(∥x∥)}{{∥x∥}^{2}}S\left(x\right)+\frac{∥x∥-\sin(∥x∥)}{{∥x∥}^{3}}S^{2}\left(x\right),\\ J_{r}\left(0\right) & =I_{3} \end{array}$$
